# Gut Microbiota Composition Is Causally Linked to Multiple Sclerosis: A Mendelian Randomization Analysis

**DOI:** 10.3390/microorganisms12071476

**Published:** 2024-07-19

**Authors:** Valeria Zancan, Martina Nasello, Rachele Bigi, Roberta Reniè, Maria Chiara Buscarinu, Rosella Mechelli, Giovanni Ristori, Marco Salvetti, Gianmarco Bellucci

**Affiliations:** 1 Department of Neurosciences, Mental Health and Sensory Organs, Sapienza University of Rome, 00185 Rome, Italy; 2 Neuroimmunology Unit, Istituto di Ricovero e Cura a Carattere Scientifico (IRCCS) Fondazione Santa Lucia, 00179 Rome, Italy; 3 Istituto di Ricovero e Cura a Carattere Scientifico (IRCCS) San Raffaele, 00163 Rome, Italy; 4 Department for the Promotion of Human Sciences and Quality of Life, San Raffaele Roma Open University, 00166 Rome, Italy; 5 Istituto di Ricovero e Cura a Carattere Scientifico (IRCCS) Istituto Neurologico Mediterraneo Neuromed, 86077 Pozzilli, Italy

**Keywords:** gut microbiota, multiple sclerosis, Mendelian randomization

## Abstract

Accumulating evidence links the microbial communities inhabiting the gut to the pathophysiological processes underlying multiple sclerosis (MS). However, most studies on the microbiome in MS are correlative in nature, thus being at risk of confounding and reverse causality. Mendelian randomization (MR) analyses allow the estimation of the causal relationship between a risk factor and an outcome of interest using genetic variants as proxies for environmental exposures. Here, we performed a two-sample MR to assess the causality between the gut microbiome and MS. We extracted genetic instruments from summary statistics from three large genome-wide association studies (GWASs) on the gut microbiome (18,340, 8959, and 7738 subjects). The exposure data were derived from the latest GWAS on MS susceptibility (47,429 patients and 68,374 controls). We pinpointed several microbial strains whose abundance is linked with enhanced MS risk (*Actinobacteria* class, *Bifidobacteriaceae* family, *Lactobacillus* genus) or protection (*Prevotella* spp., *Lachnospiranaceae* genus, *Negativibacillus* genus). The largest risk effect was seen for *Ruminococcus Torques* (OR, 2.89, 95% C.I. 1.67–5, *p* = 1.51 × 10^−4^), while *Akkermansia municiphila* emerged as strongly protective (OR, 0.43, 95% C.I. 0.32–0.57, *p* = 1.37 × 10^−8^). Our findings support a causal relationship between the gut microbiome and MS susceptibility, reinforcing the relevance of the microbiome–gut–brain axis in disease etiology, opening wider perspectives on host–environmental interactions for MS prevention.

## 1. Introduction

Multiple sclerosis (MS) is a chronic inflammatory and neurodegenerative disease of the central nervous system (CNS), commonly diagnosed in young adults, leading to physical disability and cognitive impairment. The causes of MS remain unclear, but many genetic and environmental factors (such as low vitamin D levels, obesity, tobacco smoking, and, most importantly, Epstein–Barr virus infections) have been pointed out as risk factors, supporting the complex nature of MS etiology [1,2]. 

During the past decade, there has been great scientific excitement around the gut microbiome in human health and disease, with special regard to inflammatory and autoimmune conditions, including multiple sclerosis [3]. 

Alterations in the gut microbiota pleiotropically impact immune function and the course of neuroinflammatory diseases such as MS. First, the microbiome regulates the host’s B cell and T cell maturation and activity: briefly, a healthy microbiota promotes immune homeostasis through the release of Treg cells and IgA antibodies in the bloodstream, while dysbiosis enhances neuroinflammation through the boosting of Th17 cell differentiation. In turn, these subsets shape microbiota composition through maintenance of the intestinal barrier, and they keep microbial translocation at a low grade to other body sites, defining a scenario characterized by a constant bidirectional interaction of crucial importance [4]. Additionally, the composition of the gut microbiota influences the production of serotonin in the gut, which, in turn, impacts the systemic serotonin-mediated regulation of immune subsets.

The evidence of an association between gut microbiota alterations and MS relies on a broad literature [5,6,7,8,9]. A first study performed metagenomic analyses on the gut microbiome of 71 patients and 71 controls, identifying specific bacterial taxa which were significantly enriched in patients with MS (*Akkermansia muciniphila* and *Acinetobacter calcoaceticus*), while others were reduced (*Parabacteroides distasonis*). Fecal transplants from patients with MS into germ-free mice resulted in more severe symptoms of experimental autoimmune encephalomyelitis and reduced proportions of IL-10+ Tregs, compared to control-derived transplants [10]. Breakthrough evidence recently came from a large multi-center study in which the gut microbiome of 576 patients with MS and household healthy controls (1152 total subjects) was analyzed, highlighting some species which were significantly increased (*Akkermansia muciniphila*, *Ruthenibacterium lactatiformans*, *Hungatella hathewayi*, and *Eisenbergiella tayi*) and other which were significantly decreased (*Faecalibacterium prausnitzii* and *Blautia* species) in patients with MS. The authors related these gut microbiome alterations to both MS risk and MS course and progression. Furthermore, distinct microbe–microbe interactions and metabolic pathways were found in patients with MS compared to the healthy controls (HCs), and the gut microbiome’s modulation with a disease-modifying therapy was pointed out [5].

A limit of most published studies on the microbiome in MS is their observational design, which is correlative in nature and, thus, intrinsically prone to confounding factors and reverse causality [11]. Mendelian randomization (MR) represents a novel epidemiological study design, which has become increasingly common since the broad diffusion of genome-wide association studies. MR aims to assess the causal association between an environmental risk factor (exposure) and a disease (outcome) [11]. Unlike clinical randomized trials, in which the two study groups are determined by random assignment to an exposure factor (cases) or not (controls), in MR studies, the two groups are defined in function of a genetic variant (called instrumental variable, IV), assuming that randomization relies on Mendel’s law of the random assortment of genetic variants. Instrumental variables rely on single-nucleotide polymorphisms (SNPs) strongly associated with exposure, representing a proxy of exposure to infer the causal association between this and the disease [12]. Given their genetic nature, IVs are independent from confounders that may influence exposure and outcome [13]. 

In this study, a two-sample MR was conducted to assess the causal link between microbiome composition and MS risk. Our aim was to strengthen the existing evidence on dysbiosis as a risk factor for MS by performing an MR analysis, intrinsically able to confer a causal meaning to an association between an exposure and a disease.

## 2. Materials and Methods

With respect to data sources, exposure data were extracted from summary statistics of three large GWAS assessing the influence of host genetics on gut microbiome composition:

(1) The most comprehensive study in this domain, by the MiBioGen consortium, in which 16S rRNA sequencing profiles were collected from 24 cohorts, for a total of 18,340 individuals [14];
(2) The work by Qin and colleagues, in which metagenomic sequencing was performed in a single population-based cohort of 5959 subjects [15];
(3) The work by Lopera-Maya and colleagues, which assessed SNP-to-taxon and SNP-to-microbial function associations from stool shotgun metagenomic studies of 7738 participants in the Dutch Microbiome Project [16].

Outcome data were extracted from summary statistics of the 2019 IMSGC GWAS on MS susceptibility, performed on 47,429 people with MS and 68,374 controls [17].

Regarding data analysis, we selected SNPs associated with exposure with a *p*-value threshold of 1 × 10^−8^. For IVs, we retained independent SNPs performing clumping with a linkage disequilibrium (LD) threshold of 0.001 and a 10 Mb clumping window (using the 1000 Genomes European data as a reference). SNPs with an F-statistic below 10, indicating insufficient strength, were excluded [18]. Data harmonization was performed between the gut and MS datasets, and SNPs with a minor allele frequency (MAF) ≤ 0.01, ambiguities, and palindromes were excluded.

MR causality tests were assessed using Wald’s ratio, and, where possible (i.e., with multiple IVs for the same exposure), we pooled the Wald ratios through a meta-analysis, using the inverse-variance-weighted (IVW) method. To exclude reverse causality, we used the MR Steiger directionality test [19].

A nominal *p*-value of 0.05 was used as the threshold for statistical significance. MR analyses were conducted using the TwoSampleMR package [20] in the R software (version 4.2.1).

## 3. Results

**Figure 1:**
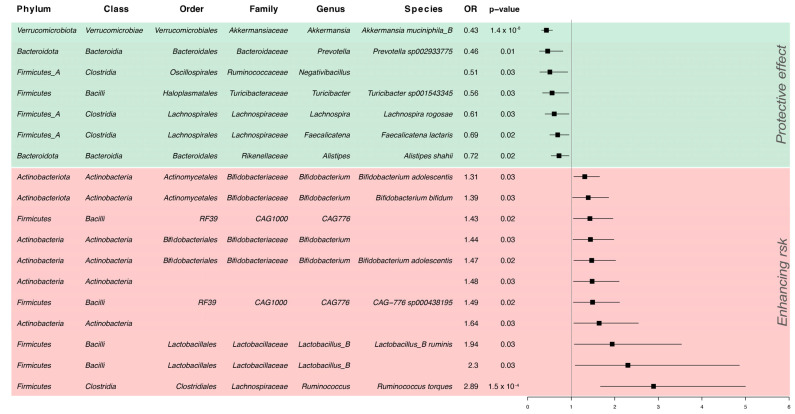
Forest plot of significant associations from MR analysis on gut microbiota composition and MS susceptibility. Protective strains (OR < 1) are shown in green and risk-enhancing strains in red (OR > 1). OR: odds ratio. The complete results are available in Supplementary Table S1.

We performed two-sample MR analyses to explore the causal link between gut microbial composition and MS. Following stringent selection criteria and after harmonization, we held a total of 123 IVs for analysis. Figure 1 summarizes the statistically significant results (see Supplementary Table S1 for the complete results).

We observed a consistently positive causal effect of species from the *Actinobacteria* class on MS risk. Among the *Bifidobacteriaceae* family, an increase of one standard deviation (SD) in the abundance of *Bifidobacterium adolescentis* was associated with a 31–47% increased risk of MS, consistent in two different association sources. A similar effect was seen for *Bifidobacterium bifidum* abundance (OR, 1.39; 95% C.I. 1.03–1.86, *p* = 0.03).

Also, the *Lactobacillus_B* genus (OR, 2.30; 95% C.I. 1.09–4.86, *p* = 0.03) and the relative species *Ruminis* from the same genus (OR, 1.94, 95% C.I. 1.07–3.53, *p* = 0.03) were positively associated with MS.

The strongest causal association was seen for the species *Ruminococcus Torques* (O.R. 2.89, 95% C.I. 1.67–5, *p* = 1.51 × 10^−4^), pertaining to the *Clostridia* class.

Conversely, we found a negative association between the abundance of *Prevotella* and MS: an increase of one standard deviation ( SD) in the abundance of *Prevotella* sp002933775 corresponded to a 54% reduction in MS risk. Other microbial strains from the *Clostridia* class appeared to be protective: *Lachnospira rogosae* (OR, 0.61, 95% C.I. 0.40–0.95, *p* = 0.03), *Faecalicatena lactaris* from the *Lachnospiranaceae* genus (OR, 0.51, 95% C.I. 0.51–0.95, *p* = 0.02), and the *Negativibacillus* genus from *Ruminococcaceae* (OR, 0.51, 95% C.I. 0.28–0.92, *p* = 0.03). Another protective signal came from a *Turibacter* strain from *Firmicutes* (OR, 0.56, 95% C.I. 0.34–0.94, *p* = 0.03).

The strongest protective association was seen for the species *Akkermansia Municiphila_B* (OR, 0.43, 95% C.I. 0.32–0.57, *p* = 1.37 × 10^−8^).

Steiger’s test confirmed, for all associations, the correct directionality, i.e. the exposure (microbial strain abundance) causes the outcome (MS) (*p* < 0.05 for all IVs). 

## 4. Discussion

This two-sample MR study pinpointed that the relative abundance of *Ruminococcus torques*, *Actinobacteria*, *Bifidobacterium adolescentis*, *Bifidobacterium bifidum,* and *Lactobacillus* enhanced MS risk, while *Akkermansia muciniphila* and *Prevotella* exerted protective effects. Additionally, our analysis highlighted protective signals from commonly understudied bacterial strains, such as the *Negativibacillus* genus, *Lachnospira rogosae* and *Faecalicatema* lactaris—components of the *Lachnospiraceae* family—*Turibacter*, and *Alisteps shahii*.

We found that the genetically predicted abundance of *Ruminococcus torques* is causally linked to MS. Our finding converges with evidence from the large multi-center International Multiple Sclerosis Microbiome Study (iMSMS), which found this bacterium to be enriched in untreated patients with MS [5]. Furthermore, the bacterial expansion of the genus *Ruminococcus* has also been highlighted during experimental autoimmune encephalomyelitis (EAE), a well-accepted animal model for MS [21]. Finally, *Ruminococcus* abundance was found to decrease in patients with MS following treatment with DMTs (interferon beta1a or teriflunomide) [22]. *Ruminococcus* spp., including *R. torques*, are abundant in the gastrointestinal tract, endowed with diverse metabolic functions, and are potent mucus degraders [23]; still, a mechanistic interpretation of *Ruminococcus torques’* role in MS is far from being revealed.

We found causal links between MS and the abundance of *Bifidobacterium adolescentis*, *Bifidobacterium bifidum,* and, higher in the taxonomic hierarchy, the *Actinobacteria* class. Intriguingly, a recent work unraveling the gut microbiota of pediatric MS cases highlighted an MS-specific enrichment of both *Actinobacteria*, at the class level, and *Bifidobacterium*, at the genus level [24]. Similarly, in an Egyptian cohort of patients with MS, an increase in *Actinobacteria* was found compared to the healthy controls [25]. Looking at the underlying pathobiology, there is some evidence regarding the ability of both *Bifidobacterium adolescentis* [26] and *Bifidobacterium bifidum* [27] to boost Th17 cell differentiation, potentially enhancing neuroinflammation. Conversely, other works have suggested anti-inflammatory effects of these strains, which might be exerted through metabolic modulation and induction of Treg differentiation [28]. As for *R. Torques*, *Bifidobacterium adolescentis* abundance was found to be influenced by DMTs in the iMSMS study [5].

We found that the genetically predicted abundance of *Akkermansia muciniphila* represented a protective factor for MS susceptibility. At a first glance, this result could be surprising, since a relative abundance of *Akkermansia* in the gut microbiome of patients with MS is one of the most recognized patterns in studies of gut dysbiosis and MS [5,6,10,29,30]. Among the possible explanations for this is the fact that *Akkermansia muciniphila* abundance represents an adaptive, compensatory response to promote eubiosis recovery rather than an underlying pathogenic event [31]. In a recent study on the gut microbiota of patients with progressive MS, *Akkermansia* was linked to lower disability and, consistent with this, ameliorating EAE [29]. Relevant to this is the fact that the microRNA miR-30d, enriched in the feces of people with MS and animals with EAE, was shown to expand the commensal *Akkermansia muciniphila* subsets, which, in turn, promoted regulatory T cells induction and ameliorated EAE; fecal transfer from the EAE model at peak disease (i.e., after *Akkermansia* expansion) was capable to prevent disease onset in susceptible mice [32]. As for the Epstein–Barr virus, *Akkermansia* epitopes were found to elicit autoreactive CD4+ T cell clones in MS in association with the HLA-DR15 risk haplotype, driving cross-reactive neuropathology through molecular mimicry [33,34]. A similar pattern has been found for the humoral response: increased levels of IgG against *A. muciniphila* were found in the CSF of patients with RRMS compared to the controls [35]. Our results may also suggest that an increased abundance of *A. municiphila* antigens before disease onset may favor immune tolerance or ignorance and limit an efficient, antigen-specific, and potentially cross-reactive response.

Finally, worth mentioning is *Prevotella’s* protective effect on MS. In observational studies, *Prevotella* is frequently found to be reduced in patients with MS compared to the healthy controls [36,37,38]. In the iMSMS study, in the part dealing with interacting microbial communities defining MS and healthy control (HCC) networks, *Prevotella* emerged among the 45 species enriched in the healthy controls [5]. Among the *Prevotella* species, *Prevotella histicola* has been linked to an improved EAE course [39]. Contrariwise, there are studies highlighting a possible proinflammatory role of *Prevotella* strains [40]. Further experimental evidence is needed to test such an association.

A limitation of this study is that, to keep stringent IV selection criteria, associations are supported by single IVs, possibly influencing precision. However, the consistency of the results coming from different exposure sources, performed through different genotyping techniques, supports our findings. In the future, a larger sample size for the microbial GWAS could ensure greater power for detecting genetic and epidemiological links to complex diseases, especially with preventive aims. Additionally, to date, generalizability to genetic ancestries other than European is limited, and cross-ancestry efforts for both microbiota and disease mapping are awaited. 

Furthermore, knowledge on the genetic influence of microbial composition in body sites other than the gut is extremely limited to date and is awaited to extend our view on host–microbiome interactions in health and disease.

## 5. Conclusions

Our findings provide evidence for a causal relationship between some gut microbiota strains and MS risk, reinforcing the relevance of the microbiome–gut–brain axis in disease etiology and the crucial importance of host–environmental interactions in MS. Targeted modulation of the gut microbiota may hold promise for the development of novel therapeutic strategies aimed at mitigating MS risk.

## Data Availability

Publicly available datasets were analyzed in this study.

## Supplementary Materials

The following supporting information can be downloaded at https://www.mdpi.com/article/10.3390/microorganisms12071476/s1: Table S1: Full results from the MR analysis. References [14,15,16] are cited in the supplementary materials.

## References

[1] McGinley M.P., Goldschmidt C.H., Rae-Grant A.D. (2021). Diagnosis and Treatment of Multiple Sclerosis: A Review. JAMA—J. Am. Med. Assoc..

[2] Aloisi F., Giovannoni G., Salvetti M. (2023). Review Epstein-Barr Virus as a Cause of Multiple Sclerosis: Opportunities for Prevention and Therapy. Lancet Neurol..

[3] Wang X., Liang Z., Wang S., Ma D., Zhu M., Feng J. (2022). Role of Gut Microbiota in Multiple Sclerosis and Potential Therapeutic Implications. Curr. Neuropharmacol..

[4] Correale J., Hohlfeld R., Baranzini S.E. (2022). The Role of the Gut Microbiota in Multiple Sclerosis. Nat. Rev. Neurol..

[5] Zhou X., Baumann R., Gao X., Mendoza M., Singh S., Katz Sand I., Xia Z., Cox L.M., Chitnis T., Yoon H. (2022). Gut Microbiome of Multiple Sclerosis Patients and Paired Household Healthy Controls Reveal Associations with Disease Risk and Course. Cell.

[6] Berer K., Gerdes L.A., Cekanaviciute E., Jia X., Xiao L., Xia Z., Liu C., Klotz L., Stauffer U., Baranzini S.E. (2017). Gut microbiota from multiple sclerosis patients enables spontaneous autoimmune encephalomyelitis in mice. Proc. Natl. Acad. Sci. USA.

[7] Farshbafnadi M., Agah E., Rezaei N. (2021). The Second Brain: The Connection between Gut Microbiota Composition and Multiple Sclerosis. J. Neuroimmunol..

[8] Mirza A., Mao-Draayer Y. (2017). The gut microbiome and microbial translocation in multiple sclerosis. Clin. Immunol..

[9] Pröbstel A.K., Baranzini S.E. (2018). The Role of the Gut Microbiome in Multiple Sclerosis Risk and Progression: Towards Characterization of the “MS Microbiome”. Neurotherapeutics.

[10] Cekanaviciute E., Yoo B.B., Runia T.F., Debelius J.W., Singh S., Nelson C.A., Kanner R., Bencosme Y., Lee Y.K., Hauser S.L. (2017). Gut Bacteria from Multiple Sclerosis Patients Modulate Human T Cells and Exacerbate Symptoms in Mouse Models. Proc. Natl. Acad. Sci. USA.

[11] Allman P.H., Aban I.B., Tiwari H.K., Cutter G.R. (2018). An Introduction to Mendelian Randomization with Applications in Neurology. Mult. Scler. Relat. Disord..

[12] Xu Q., Ni J.J., Han B.X., Yan S.S., Wei X.T., Feng G.J., Zhang H., Zhang L., Li B., Pei Y.F. (2022). Causal Relationship Between Gut Microbiota and Autoimmune Diseases: A Two-Sample Mendelian Randomization Study. Front. Immunol..

[13] Xiang K., Wang P., Xu Z., Hu Y.Q., He Y.S., Chen Y., Feng Y.T., Yin K.J., Huang J.X., Wang J. (2021). Causal Effects of Gut Microbiome on Systemic Lupus Erythematosus: A Two-Sample Mendelian Randomization Study. Front. Immunol..

[14] Kurilshikov A., Medina-Gomez C., Bacigalupe R., Radjabzadeh D., Wang J., Demirkan A., Le Roy C.I., Raygoza Garay J.A., Finnicum C.T., Liu X. (2021). Large-scale association analyses identify host factors influencing human gut microbiome composition. Nat. Genet..

[15] Qin Y., Havulinna A.S., Liu Y., Jousilahti P., Ritchie S.C., Tokolyi A., Sanders J.G., Valsta L., Brożyńska M., Zhu Q. (2022). Combined effects of host genetics and diet on human gut microbiota and incident disease in a single population cohort. Nat. Genet..

[16] Lopera-Maya E.A., Kurilshikov A., Van Der Graaf A., Hu S., Andreu-sánchez S., Chen L., Vila A.V., Gacesa R., Sinha T., Collij V. (2022). Effect of Host Genetics on the Gut Microbiome in 7,738 Participants of the Dutch Microbiome Project. Nat. Genet..

[17] Nafee T., Watanabe R., Fregni F., Fregni F. (2018). Multiple Sclerosis. Clinical Trials in Neurology.

[18] Pierce B.L., Ahsan H., Vanderweele T.J. (2011). Power and Instrument Strength Requirements for Mendelian Randomization Studies Using Multiple Genetic Variants. Int. J. Epidemiol..

[19] Hemani G., Tilling K., Smith G.D. (2017). Orienting the Causal Relationship between Imprecisely Measured Traits Using GWAS Summary Data. PLoS Genet..

[20] Hemani G., Zheng J., Elsworth B., Wade K.H., Haberland V., Baird D., Laurin C., Burgess S., Bowden J., Langdon R. (2018). The MR-Base Platform Supports Systematic Causal Inference across the Human Phenome. eLife.

[21] Johanson D.M., Goertz J.E., Marin I.A., Costello J., Overall C.C., Gaultier A. (2020). Experimental Autoimmune Encephalomyelitis Is Associated with Changes of the Microbiota Composition in the Gastrointestinal Tract. Sci. Rep..

[22] Vacaras V., Muresanu D.F., Buzoianu A.D., Nistor C., Vesa S.C., Paraschiv A.C., Botos-Vacaras D., Vacaras C., Vithoulkas G. (2023). The Role of Multiple Sclerosis Therapies on the Dynamic of Human Gut Microbiota. J. Neuroimmunol..

[23] Rajilić-Stojanović M., de Vos W.M. (2014). The First 1000 Cultured Species of the Human Gastrointestinal Microbiota. FEMS Microbiol. Rev..

[24] Tremlett H., Fadrosh D.W., Faruqi A.A., Zhu F., Hart J., Roalstad S., Graves J., Lynch S., Waubant E., Aaen G. (2016). Gut Microbiota in Early Pediatric Multiple Sclerosis: A Case−control Study. Eur. J. Neurol..

[25] Elgendy S.G., Abd-Elhameed R., Daef E., Mohammed S.M., Hassan H.M., El-Mokhtar M.A., Nasreldein A., Khedr E.M. (2021). Gut Microbiota in Forty Cases of Egyptian Relapsing Remitting Multiple Sclerosis. Iran. J. Microbiol..

[26] Tan T.G., Sefik E., Geva-Zatorsky N., Kua L., Naskar D., Teng F., Pasman L., Ortiz-Lopez A., Jupp R., Wu H.J.J. (2016). Identifying Species of Symbiont Bacteria from the Human Gut That, Alone, Can Induce Intestinal Th17 Cells in Mice. Proc. Natl. Acad. Sci. USA.

[27] López P., Gueimonde M., Margolles A., Suárez A. (2010). Distinct Bifidobacterium Strains Drive Different Immune Responses in Vitro. Int. J. Food Microbiol..

[28] Toghi M., Bitarafan S., Kasmaei H.D., Ghafouri-Fard S. (2019). Bifidobacteria: A Probable Missing Puzzle Piece in the Pathogenesis of Multiple Sclerosis. Mult. Scler. Relat. Disord..

[29] Cox L.M., Maghzi A.H., Liu S., Tankou S.K., Dhang F.H., Willocq V., Song A., Wasén C., Tauhid S., Chu R. (2021). Gut Microbiome in Progressive Multiple Sclerosis. Ann. Neurol..

[30] Jangi S., Gandhi R., Cox L.M., Li N., Von Glehn F., Yan R., Patel B., Mazzola M.A., Liu S., Glanz B.L. (2016). Alterations of the Human Gut Microbiome in Multiple Sclerosis. Nat. Commun..

[31] Cani P.D., Depommier C., Derrien M., Everard A., de Vos W.M. (2022). Akkermansia Muciniphila: Paradigm for next-Generation Beneficial Microorganisms. Nat. Rev. Gastroenterol. Hepatol..

[32] Liu S., Rezende R.M., Moreira T.G., Tankou S.K., Cox L.M., Wu M., Song A., Dhang F.H., Wei Z., Costamagna G. (2019). Oral Administration of miR-30d from Feces of MS Patients Suppresses MS-like Symptoms in Mice by Expanding Akkermansia Muciniphila. Cell Host Microbe.

[33] Martin R., Sospedra M., Eiermann T., Olsson T. (2021). Multiple Sclerosis: Doubling down on MHC. Trends Genet..

[34] Wang J., Jelcic I., Mühlenbruch L., Haunerdinger V., Toussaint N.C., Zhao Y., Cruciani C., Faigle W., Naghavian R., Foege M. (2020). HLA-DR15 Molecules Jointly Shape an Autoreactive T Cell Repertoire in Multiple Sclerosis. Cell.

[35] Vallino A., Dos Santos A., Mathé C.V., Garcia A., Morille J., Dugast E., Shah S.P., Héry-Arnaud G., Guilloux C.A., Gleeson P.J. (2020). Gut Bacteria Akkermansia Elicit a Specific IgG Response in CSF of Patients with MS. Neurol. Neuroimmunol. NeuroInflammation.

[36] Chen J., Chia N., Kalari K.R., Yao J.Z., Novotna M., Soldan M.M.P., Luckey D.H., Marietta E.V., Jeraldo P.R., Chen X. (2016). Multiple Sclerosis Patients Have a Distinct Gut Microbiota Compared to Healthy Controls. Sci. Rep..

[37] Mirza A., Forbes J.D., Zhu F., Bernstein C.N., Van Domselaar G., Graham M., Waubant E., Tremlett H. (2020). The Multiple Sclerosis Gut Microbiota: A Systematic Review. Mult. Scler. Relat. Disord..

[38] Ordoñez-Rodriguez A., Roman P., Rueda-Ruzafa L., Campos-Rios A., Cardona D. (2023). Changes in Gut Microbiota and Multiple Sclerosis: A Systematic Review. Int. J. Environ. Res. Public Health.

[39] Shahi S.K., Jensen S.N., Murra A.C., Tang N., Guo H., Gibson-corley K.N., Zhang J., Karandikar N.J., Murray J.A., Mangalam A.K. (2020). Human Commensal Prevotella Histicola Ameliorates Disease as Effectively as Interferon-Beta in the Experimental Autoimmune Encephalomyelitis. Front. Immunol..

[40] Larsen J.M. (2017). The Immune Response to Prevotella Bacteria in Chronic Inflammatory Disease. Immunology.

